# The CTSA program’s role in improving rural public health: Community-engaged disease prevention and health care innovation

**DOI:** 10.1017/cts.2020.541

**Published:** 2020-09-11

**Authors:** Xinzhi Zhang, Michael G. Kurilla, Christopher P. Austin

**Affiliations:** 1 Division of Clinical Innovation, National Center for Advancing Translational Sciences, National Institutes of Health, Bethesda, MD, USA; 2 Office of the Director, National Center for Advancing Translational Sciences, National Institutes of Health, Bethesda, MD, USA

## Introduction

Rural–urban health inequities have been recognized and extensively documented for several decades [1–4]. In recent years, changing demographics and emerging epidemics including the opioid crisis and COVID-19 pandemic have further underscored the need to address health care access and quality disparities facing rural populations. Socioeconomic constraints, health care provider shortages, and a lack of health insurance contribute to higher mortality rates relative to their urban counterparts [5]. Although certain barriers can be addressed at federal or state levels, regional or community-level interventions including preventive practice and early disease detection and treatment may be more effective and efficient to improve rural health outcomes in a timely manner [6]. The Secretary of Health and Human Services (HHS) identified rural health as a Department priority and established the Rural Health Task Force in late 2018. The goal of the task force is to bring together disparate efforts across HHS and develop the best understanding of where policy and program changes can effect transformation.

In the fiscal year 2019, the US Congress included report language with the National Center for Advancing Translational Sciences (NCATS) appropriation that requested the Clinical and Translational Science Awards (CTSA) program to apply the NCATS translational science paradigm to the problem of rural health disparities. The CTSA program supports a national network of medical research institutions that work together to improve the translational research process with the goal of getting more diagnoses and treatments to more patients more quickly. The CTSA institutions (called “hubs”) collaborate locally and regionally to catalyze innovation in translational science research, processes, and training, and provide resources and opportunities to develop innovative approaches and technologies to make translation more efficient and effective. Program support and collaborative initiatives harmonize efforts, foster cross-hub collaboration, and strengthen the CTSA network to improve the quality, safety, efficiency, and speed of clinical and translational research nationally.

## Public Health Needs and Research Gaps in Rural America

Nonmetropolitan areas have higher age-adjusted death rates from the five leading causes of death than metropolitan areas including heart disease, stroke, cancer, unintentional injury, and chronic lower respiratory disease, which makes the rural population more susceptible to SARS-CoV-2 [7]. Age-adjusted prevalence of chronic obstructive pulmonary disease (COPD), hospitalizations, and deaths caused by COPD was also higher among rural residents than residents living in micropolitan or metropolitan areas [8]. In recent years, age-adjusted premature death rates have increased among rural low socioeconomic status Whites, mostly attributable to suicide, poisoning, and liver disease [9]. The Centers for Disease Control and Prevention (CDC) reported that drug overdose death rates are significantly higher in rural than in urban areas [10]. The CDC identified 220 US counties, the majority rural, that are highly vulnerable to both HIV and hepatitis C virus (HCV) infections [11]. Among rural counties, almost all (91%–99%) lack an opioid treatment program facility and 72% did not have a physician with a waiver to prescribe or dispense buprenorphine for opioid use disorder treatment [12].

As of September 2017, 60% of Health Professional Shortage Areas, as identified by the Department of Health & Human Services, were in rural America; an estimated 22.2 million rural residents reside in these areas. In rural America, financing of health care and health insurance coverage are heavily dependent on Medicare and Medicaid [13]. Disproportionately high reliance on public funding in these geographic areas has significant implications for health inequities. For example, while American Indians experience some of the greatest health disparities, they report significantly lower use of Medicaid-paid ambulatory visits, prescriptions, emergency room visits, and hospitalizations compared with Whites [14]. In a national survey of rural health stakeholders, access to emergency services, primary care, and health insurance was reported as high-priority needs [15]. Indeed, more than 20% of rural hospitals, mostly in the South (including Alabama, Georgia, Oklahoma, Texas, Kansas, Mississippi, and Tennessee), were predicted to be at high or mid-high risk of financial distress in 2019 [16]. Communities served by at-risk rural hospitals usually have larger percentages of non-Whites, lower socioeconomic status, and worse health outcomes than their counterparts. Payer mix degradation, declining inpatient care, and inability to leverage innovation have been major factors driving the rural hospital crisis [17].

Meanwhile, rural populations are becoming more diverse and older with increasing African American and Hispanic populations and a higher proportion of older adults [15]. More than 20% of all rural residents are racial/ethnic minorities. However, there has been a notable lack of research on intra-rural differences in health by race and ethnicity [18]. Racial/ethnic minorities may face different challenges in rural areas in addition to the lack of health-care access, including discrimination and mistrust of health care institutions due to historical factors, recent immigration experiences, and isolation. A recent CDC Report examined racial/ethnic health disparities in rural areas of the USA and found that African Americans, Hispanics, and American Indians/Alaska Natives were more likely than their White counterparts to report their health as fair or poor, that they were obese, and that they were unable to access health care because of cost [19]. Also, beliefs about the behavioral determinants of health may differ between urban and rural populations or by geographic region. For example, Appalachian as compared to non-Appalachian respondents to the Health Information National Trends Survey (2011–2014) reported being less likely to believe that lifestyle factors were related to obesity [20].

## Community-Engaged Disease Prevention and Health Care Innovation at CTSA Hubs

The National Academy of Science’s 2005 “Quality through Collaboration: The Future of Rural Health” report identified five strategies to address the quality of health care chasm in rural communities. Five strategies include: (1) Adopt an integrated, prioritized approach to addressing both personal and population health needs at the community level; (2) Establish a stronger quality improvement support structure to assist rural health systems and professionals in acquiring knowledge and tools to improve quality; (3) Enhance the human resource capacity of rural communities, including the education, training, and deployment of health care professionals, and the preparedness of rural residents to engage actively in improving their health and health care; (4) Monitor rural health-care systems to ensure that they are financially stable and provide assistance in securing the necessary capital for system redesign; and (5) Invest in building an Information Communications Technology infrastructure, which has enormous potential to enhance health and health care over the coming decades. However, after a decade, many structural and financial barriers, especially the digital divide between rural and urban communities, remain and warrant further public health attention. Most recently, the COVID-19 pandemic has shown the importance and value of telemedicine in access to health care.

Addressing health disparities has always been a place-based issue [21]. In April 2017, the White House issued an executive order establishing the Interagency Task Force on Agriculture and Rural Prosperity “to ensure the informed exercise of regulatory authority that impacts agriculture and rural communities.” For example, residents of colonias near the Texas-Mexico border – predominantly Latino, unincorporated, and impoverished – continue to live without running water or electricity or education opportunities [15]. Women who live in rural areas experience disproportionately high mortality rates [22]. Behavioral risk factors associated with obesity and smoking contribute to rural–urban inequities throughout the country [23]. Multilevel and multicomponent interventions through transdisciplinary teams are needed to translate evidence-based preventive practice into culturally, linguistically, and geographically adapted interventions in rural communities [24].

Since 2006, the CTSA program has focused on a number of major goals to advance clinical and translational science: innovate processes to increase the quality and efficiency of translational research; engage patients and communities in every phase of the translational process; promote the integration of special and underserved populations in translational research across the human lifespan; train and cultivate the translational science workforce; and advance the use of informatics and data science in translation. These goals align well with the national rural health strategies set forth by the US Department of Health and Human Services. In April 2019, a CTSA sponsored event on Rural Health and Health Equity was hosted by the University of Florida Clinical and Translational Science Institute to catalyze new collaborations among the translational science, cooperative extension, and other sectors to improve rural health and achieve health equity. Recently, a new website was released that highlights the CTSA program’s role and activities in improving rural public health translation.

### Health Care and Access

As a network of 60 leading academic medical institutions, the CTSA program has a scientific, medical, and moral responsibility to lead in building a more efficient, effective, and equitable health-care system for rural Americans. The increasing hospital closures in rural America and the devastating lessons of the COVID-19 pandemic call for a novel and nontraditional approach to address “structural urbanism” – a health care financing bias affecting rural residents [25]. Claire O’Hanlon and coauthors compare the attributes of the 306 rural hospitals that created health system affiliations in 2009 and 17 with those of rural hospitals that did not. They found that operating margins increased significantly following rural hospital affiliation with a health system [26]. However, affiliating hospitals “experienced a significant reduction in on-site diagnostic imaging technologies, the availability of obstetric and primary care services, and outpatient nonemergency visits.” Clearly, maintaining an equivalent quality of care while ensuring financial sustainability must be a critical priority to advance rural health equity.

Because primary care provides the majority of health care to rural populations, it is critical when designing interventions to engage primary care practices that serve people in rural communities. For example, Project ECHO (Extension for Community Healthcare Outcomes) at the University of New Mexico can connect academic medical institutions with extensive resources and specialist expertise to community providers, and provide remote support for the diagnosis and treatment of complicated conditions such as HCV infection [27]. Moreover, ECHO provides excellent opportunities for peer-to-peer learning and contributes to medical education in rural and underserved areas. The US Centers for Medicare and Medicaid Services (CMS) have also tested innovative models of integrated, coordinated health care in rural areas including Pennsylvania Rural Health Model and Rural Community Hospital Demonstration. The Pennsylvania Rural Health Model, for example, has set three population health targets including increased access to primary and specialty care, decreased rural health disparities through improved chronic disease management, and fewer deaths from substance use [28]. Dissemination and implementation of such programs across the CTSA network could benefit rural residents in many remote areas.

### Technology and Innovation

It is critical to identify research gaps and needed actions to improve rural health outcomes through advancing technology innovation, which includes the application of telemedicine and telehealth. According to the Office of the National Coordinator for HIT, health information technology (HIT) refers to the electronic systems health care providers and patients use to store, share, and analyze related health information [29]. Telemedicine is a method of delivering health care that makes the use of HIT to accomplish its goals, while telehealth is often used to encompass a broader application of technologies to distance education, consumer outreach, and other applications [30]. A great example is the expansion and modification of Project ECHO or similar telemedicine initiatives among CTSA institutions. In 2019, NCATS awarded $3.6 million to a telehealth network, Supporting Pediatric Research on Outcomes and Unitization of Telehealth (SPROUT), to establish an innovative and collaborative model integrating telehealth into pediatric practices. The lack of access to care and the recent closure of hospitals in rural areas have emphasized the need to expand HIT especially telemedicine for rural underserved populations. This is particularly important given that 80% of the 24 million Americans that lack access to broadband are in rural communities [31].

Meanwhile, although over a third of rural Americans do not have an Internet-enabled smart phone [31], mobile health can play a significant role in disease prevention and management with advanced technology such as 5G. For example, a CTSA research scholar from the University of Rochester Medical Center recently developed a mobile technology (mPower [32]) that allows patients with Parkinson’s disease to track and share their symptoms live with their health care providers. The app measures dexterity, balance and gait, voice, and memory multiple times each day. This offers a substantial potential for patient care and disease management. COVID-19 has demonstrated that telemedicine is practical and that rural telehealth merits expansion [33]. Culturally appropriate, promising best practices related to the advancement of health information technology, telehealth, remote patient monitoring, and collaborative partnerships to address behavioral health shortages are emerging among rural populations [34]. During the pandemic, for the first time, CDC issued guidelines on how to use telehealth to expand access to essential health services, and HHS launched its first telehealth website (Telehealth.hhs.gov). CMS and other insurance payers have evaluated different approaches to provide geographic and other flexibility during the pandemic, and grant payment parity between telehealth and in-person care.

### Prevention and Disparities

The lack of specialist access has been a common barrier for rural populations. Having one or more annual visits with both a specialist and a primary care provider was associated with a 15.9% lower preventable hospitalization rate and a 16.6% lower mortality rate, relative to having only one or more visits with a primary care provider [35]. In June 2019, the National Institutes of Health (NIH) convened the Pathways to Prevention Workshop for health equity in preventive services. A systematic review of 10 preventive services recommended by the US Preventive Services Task Force (USPSTF) found that patient navigation, telephone calls and prompts, and reminders involving lay health workers increase colorectal, breast, and cervical cancer screening rates [36]. One of the 26 recommendations made by the workshop panel suggests seeking cross-sector collaborations that incorporate the clinical care system, public health, and community-based organizations [6].

A Community and Collaboration Core is a required component of all CTSA hub awards. These cores connect researchers and trainees in CTSA institutions with community-engaged research activities and help to identify community needs and inform research priorities. Both communities and researchers participate as full partners. Programs tackling rural population needs, such as the *Community Health Coalition Development Program* at the Indiana CTSA hub and *Community Scientist Academy* at the University of Arkansas for Medical Sciences CTSA hub are available to be replicated in more CTSA hubs and elsewhere. CTSA Community Collaboration Cores also have intersections with Cooperative Extension sites of the US Department of Agriculture and the Prevention Research Centers of the CDC, to address chronic disease prevention and health promotion in their communities.

The Appalachian Translational Research Network (ATRN) is a successful research consortium anchored within CTSA hub institutions including the University of Kentucky, the Ohio State University, University of Cincinnati, Pennsylvania State University, and Wake Forest University. In addition, the CTSA hub-affiliated universities Marshall University and Ohio University, as well as an Institutional Development Award for Clinical and Translational Research (IDeA-CTR) awardee, West Virginia University, are members. With a vision of “Health Equity Across Appalachia,” the network has addressed the significant health challenges of the Appalachian population, nearly half of which lives in rural areas with limited health care access. Their fruitful community outreach and research collaboration through annual pilot studies have resulted in a 19-1 return-on-investment ratio (i.e., a 19-fold return of research investment on every dollar CTSA program invested).

### Training and Workforce

Bringing translational science innovation to the health care needs of rural populations will require a new generation of translational scientists who are not only rigorous researchers, domain experts, and team players, but also system thinkers, boundary crossers, process innovators, and skilled communicators [37]. The recent decline of medical school applicants from rural backgrounds [38] has demonstrated a critical need to reduce the workforce gap between rural and urban in order to improve rural health outcomes and reduce health inequity. For example, the Institute of Translational Health Sciences at the University of Washington CTSA hub provides early-stage investigators from WWAMI states (Washington, Wyoming, Alaska, Montana, and Idaho) with a high-quality, targeted, and structured career development package on translational science. The WWAMI region Practice and Research Network (WPRN) supports collaboration between primary care practices and academic researchers to improve the quality of care for patients in the region. The Oregon Health & Science University CTSA’s KL2 program has trained one of only two American Indian/Alaska Native (AI/AN) gynecologic oncologists in the nation for cervical cancer prevention in the Pacific Northwest AI/AN population. Moreover, the CTSA program has also established open access to a wide variety of training resources and educational materials created by CTSA hubs.

In November 2019, NIH expanded its definition of socioeconomic disadvantaged for the training portfolio, in order to diversify student and faculty populations and enhance the participation of individuals from groups that are underrepresented in the biomedical, clinical, behavioral, and social sciences (NOT-OD-20-031). Growing up in rural areas, as designated by the Health Resources and Services Administration rural health grants eligibility analyzer, is one of the seven criteria used for designation as “disadvantaged.” This revised definition will make more rural students and junior researchers eligible for diversity supplements supported by funding from NCATS and other NIH Institutes and Centers.

## Conclusion

Despite a decade of efforts since the National Academy’s report on rural health, many challenges to rural health equity remain. By placing increased and intentional emphasis on innovative translational science approaches to longstanding rural health disparities, the CTSA program aims to reduce the disproportionate burden of disease on rural populations, and thus make the vision of rural health equity a reality.
